# Impact of Cr Doping on the Structural, Optical, and Magnetic Properties of Sol–Gel-Synthesized Bi_0.80_Ba_0.10_Pr_0.10_FeO_3_ Nanopowders

**DOI:** 10.1021/acsomega.4c03032

**Published:** 2024-06-12

**Authors:** Subhash Sharma, Gabriel Rojas-George, Manish Kumar, Pawan Kumar, Rajesh Kumar, Santosh Kumar, Jesús M. Siqueiros, Oscar Raymond Herrera

**Affiliations:** † CONAHCyT-Centro de Nanociencias y Nanotecnología, Universidad Nacional Autónoma de México, Km 107 Carretera Tijuana-Ensenada, AP 14, Ensenada 22860, Baja California, Mexico; ‡ Centro de Nanociencias y Nanotecnología, Universidad Nacional Autónoma de México, Km 107 Carretera Tijuana-Ensenada, AP 14, Ensenada 22860, Baja California, Mexico; § CONAHCyT—Centro de Investigación en Materiales Avanzados, S.C., Miguel de Cervantes 120, Complejo Industrial Chihuahua, Chihuahua 31136, Chihuahua, Mexico; ∥ Experimental Research Laboratory, Department of Physics, ARSD College, University of Delhi, New Delhi 110021, India; ⊥ School of Basic and Applied Sciences, K. R. Mangalam University, Gurugram, Haryana 122103, India; # Department of Chemistry, Himachal Pradesh University, Summer Hill, Shimla-5, Shimla 171005, India

## Abstract

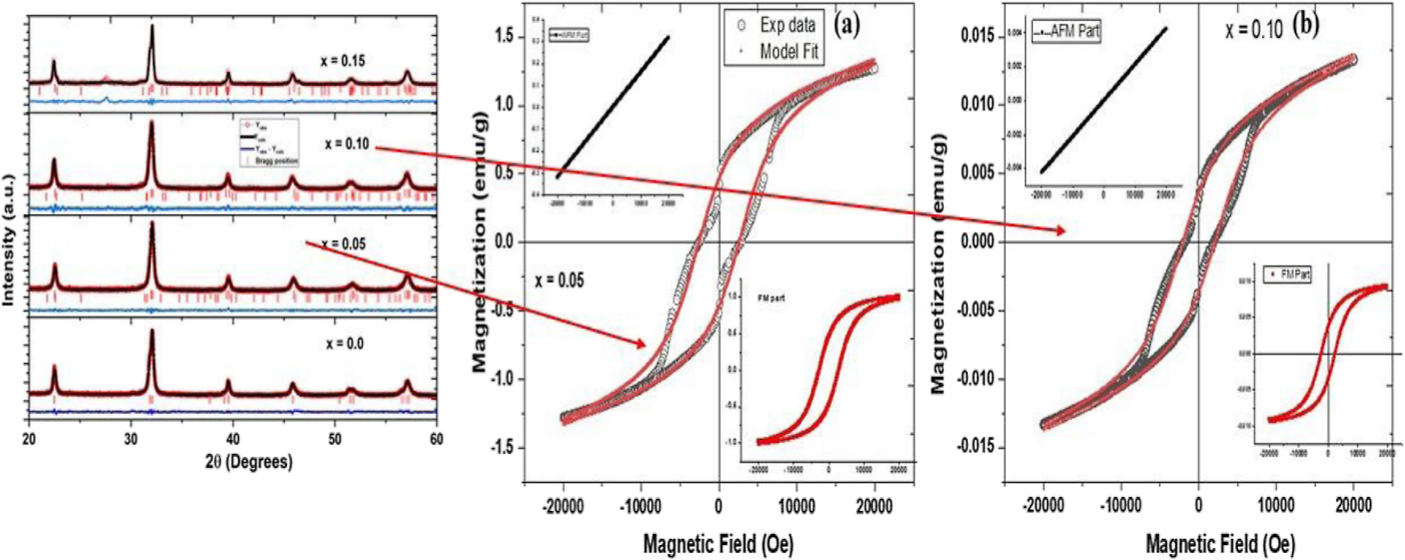

The sol–gel route was used to synthesize a series of compounds of the system Bi_0_._8_Ba_0_._10_Pr_0_._10_Fe_1–*x*_Cr_*x*_O_3_ within the 0 ≤ *x* ≤ 0.15 compositional range. To explore the impact of Cr^3+^ ion substitution on the structural, dielectric, optical, and magnetic properties, we introduced varying concentrations of Cr^3+^ while maintaining a fixed 10% atomic concentration of each Ba^2+^ and Pr^2+^ in BiFeO_3_. X-ray diffraction analysis revealed a structural phase transition from rhombohedral (*R*3*c*) for an undoped (i.e., without Cr) sample to two coexisting phases, i.e., a mix of rhombohedral and orthorhombic (*Pbnm*) phases for the Cr-doped samples. Cr^3+^ doping significantly changes the band gap energy from 1.84 eV (*x* = 0.0) to 1.93 eV (*x* = 0.15), which makes this material suitable for photovoltaic applications. Furthermore, each sample exhibited ferromagnetic behavior due to the disruption of the spiral spin structures and adjustments in superexchange interactions, attributed to modifications in the Fe–O and Fe–O–Fe bond lengths. A reduction in magnetization is observed at higher Cr concentrations that can be ascribed to the dilution of magnetic moments due to the increase of the orthorhombic phase percentage and the introduction of nonmagnetic Cr^3+^ ions. Our results show that Cr doping in the Bi_0_._8_Ba_0_._10_Pr_0_._10_FeO_3_ system induces enhanced multiferroic properties at room temperature.

## Introduction

Future technological advancements demand the development of multifunctional materials that combine diverse properties within a single crystal phase, providing new functionalities to solve current problems in the multiferroic field.^1−4^ Multiferroic (MF) materials exhibiting magnetoelectric coupling between distinct ferroic properties, such as ferroelectric and magnetic, fulfill these essential requirements.^3^ MF materials are potential candidates for various applications, including spintronic devices like magnetoelectric random access memories (MERAM), electrical switching, nanoelectronics, field-effect transistors, and sensors.^3,4^ Among multiferroic compounds, bismuth ferrite BiFeO_3_ or (BFO) stands out as a well-known candidate yet, featuring both ferroelectricity and magnetic ordering.^5,6^ The distorted rhombohedral perovskite structure with space group *R*3*c* is renowned for the intriguing coexistence of ferroelectricity (Curie temperature, *T*_c_ ∼1103 K) and antiferromagnetic ordering (Néel temperature, *T*_N_, around 643 K) over an extensive temperature range.^7,8^ However, its practical utility is hampered by nonstoichiometry, impurities, and oxygen vacancies, leading to substantial leakage current. Mitigating leakage current through ion doping and meticulous synthesis optimization is possible while enhancing its electrical and magnetic properties.^9,10^ BFO is strategically doped with various elements in diverse device applications, including rare-earth ions, lanthanides, and transition metals. The introduction of alkaline earth ions into BFO has been reported to suppress its antiferromagnetic order.^11^ Rare-earth and alkaline-earth ions often substitute for bismuth, while transition metal ions frequently substitute for iron.

Hence, extensive research has been dedicated to enhancing the magnetic and dielectric properties of BFO. Various methods have been explored to disrupt the spiral spin structure, including nanomaterials synthesis. Additionally, A-site substitutions by rare-earth elements (Ho, Er, Eu, Y, etc.) and IIA group metals (Ca, Sr, Ba, etc.) and B-site substitutions by transition elements (Mn, Co, Cr, etc.)^12−19^ have been tested. In the above-mentioned studies, the mechanism for improving the ferroelectric properties is, as expected, dilution of the A site by replacing the Bi ions. Since Bi evaporates during the sintering process and to compensate for the charge balance, induced oxygen vacancies contribute to conduction. This process reduces the ferroelectric nature of the BFO materials. Therefore, replacing Bi with another dopant reduces the possibility of evaporation and creates a more stable dipole moment compared to that when only Bi is present. Furthermore, BFO displays a spin spiral structure 62 nm in length, resulting in an AFM structure. So, changing this length by synthesizing BFO materials via a chemical route to reduce the particle size below 62 nm or/and replacing the Fe ions with other magnetic ions will increase magnetization. Structural phase transitions and changes in Fe–O–Fe angle also contribute to increased magnetization, as reported by some researchers.^1−10^ Another avenue for modification involves altering the magnetic interactions by changing the interatomic bond distances and atomic magnetic moments. Recent research has successfully demonstrated that substituting Ba^2^^+^ and Pr^3+^ ions for Bi^3+^ ions significantly enhances BFO’s magnetic properties.^18,20^ However, a separate study has revealed that substituting transition metal elements for iron in the Bi_0_._8_Ba_0.2_FeO_3_ sample decreases magnetization.^21^ A magnetic analysis is extremely important for the BiFeO_3_-based materials with doping elements supporting magnetic enhancement and ferroelectric nature. Thus, the current study focuses on enhancing the physical properties of BiFeO_3_. Recently, many researchers have investigated the impact of Cr ions on the magnetic properties of the BFO material. They found improved magnetic properties; however, the improvement is insignificant in the single Cr doping.^22−25^ So, these studies motivated us to investigate the impact of BFO-based materials. The present work with Cr doping focuses on improving the magnetic properties of BFO since Cr is a magnetic element that improves the magnetic properties to be used in applications in spintronics, magnetic storage, or sensors, given this requirement, we have fixed the codoping at the Bi site at 10 at. % of Ba and 10 at. % of Pr to stabilize the ferroelectric nature and introduced Cr^3+^ at the Fe^3+^ site to modify its magnetic properties.

## Experimental Details

The sol–gel route was adopted for synthesizing Bi_0.80_Ba_0.10_Pr_0.10_Fe_1–*x*_Cr_*x*_O_3_ for *x* = 0.0, 0.05, 0.10, and 0.15. In this route, stoichiometric amounts of all precursors were taken in nitrate form with purity >99.0% (Bi(NO_3_)_3_·5H_2_O), (Fe(NO_3_)_3_·9H_2_O), (Ba(NO_3_)_3_·9H_2_O), (Pr(NO_3_)_3_·6H_2_O), and (Cr(NO_3_)_3_·5H_2_O). First, the Bi nitrate is dissolved in deionized water; then some drops of nitric acid (64% diluted) were added and kept on a magnetic stirrer at room temperature (RT). Pr and Ba nitrates were added to the solution, followed by Fe and Cr according to the sample composition calculations. In the final step, tartaric acid was added to the solution and kept at 70 °C for 4 h. The final solution was heated at 100 °C for 12 h, followed by grinding and calcination at 600 °C for 2 h. Pelletization into disc shape was done using polyvinyl alcohol (PVA) as a binder. The sintered pellets were coated with Ag paint on both sides to measure the electrical properties and fired at 550 °C for 30 min.

## Characterization Techniques

The crystal structure and possible phase transition caused by doping were studied by X-ray diffraction (XRD) using a Panalytical X-Pert PRO diffractometer, with Cu Kα monochromatic radiation (λ = 1.5405 Å) and θ–2θ geometry. Rietveld refinement analysis was performed to obtain the lattice parameters and study the crystalline phases. The RT dielectric behavior was measured using an HP 4284A LCR meter. The optical properties of these samples were measured using a UV–vis diffuse reflectance spectrophotometer (Lamda 10, PerkinElmer). The RT magnetic properties were measured using a Physical Properties Measurement System (Quantum Design) with a VMS probe at RT (300 K).

## Results and Discussion

### Structural Analysis

**Figure 1:**
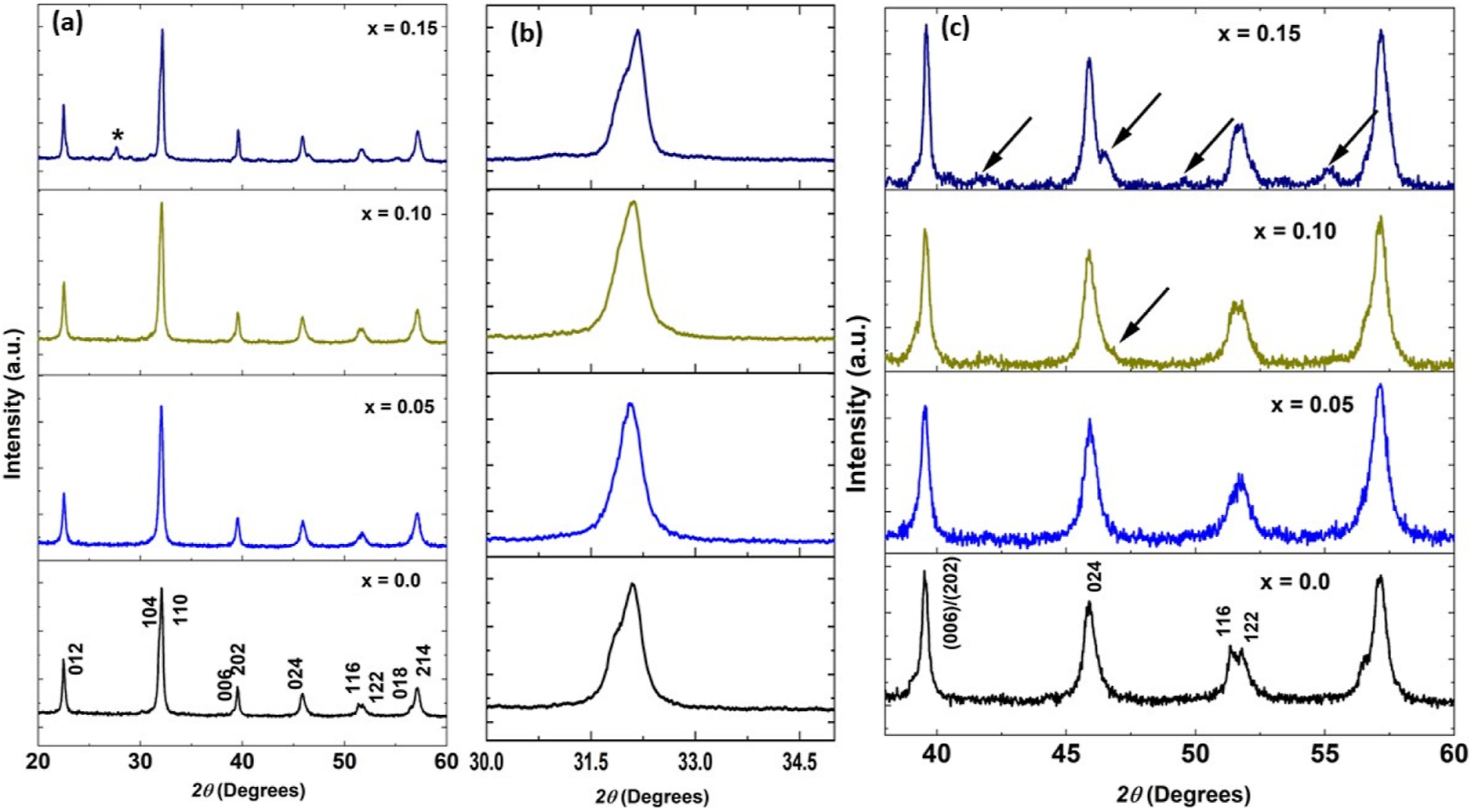
(a) XRD patterns of Cr^3+^-doped Bi_0.8_Ba_0.1_Pr_0.1_FeO_3_ samples in the composition range 0 ≤ *x* ≤ 0.15, (b) close look in the 30–35° range in 2θ, and (c) close look in the 35–60° range in 2θ.

The structural properties and possible crystalline phases were analyzed using XRD measured at RT. Figure 1a displays the XRD patterns for the studied samples. The observed peaks for undoped Bi_0.80_Ba_0.10_Pr_0.10_FeO_3_ (BBPFO) are corroborated with rhombohedral crystal structure in *R*3*c* symmetry with JCPDS file 86-1518.^26^ However, changes in the XRD patterns with Cr^3+^ incorporation are observed, indicating that a secondary phase coexists with the rhombohedral phase. On careful observation in the 31 to 33° 2θ range (see Figure 1b), the broadening and merging of peaks converting into a broadened single peak rather than two visible planes (104) and (110) suggest an apparent phase transition for all Cr^3+^-doped BBPFO samples.^27^

**Figure 2:**
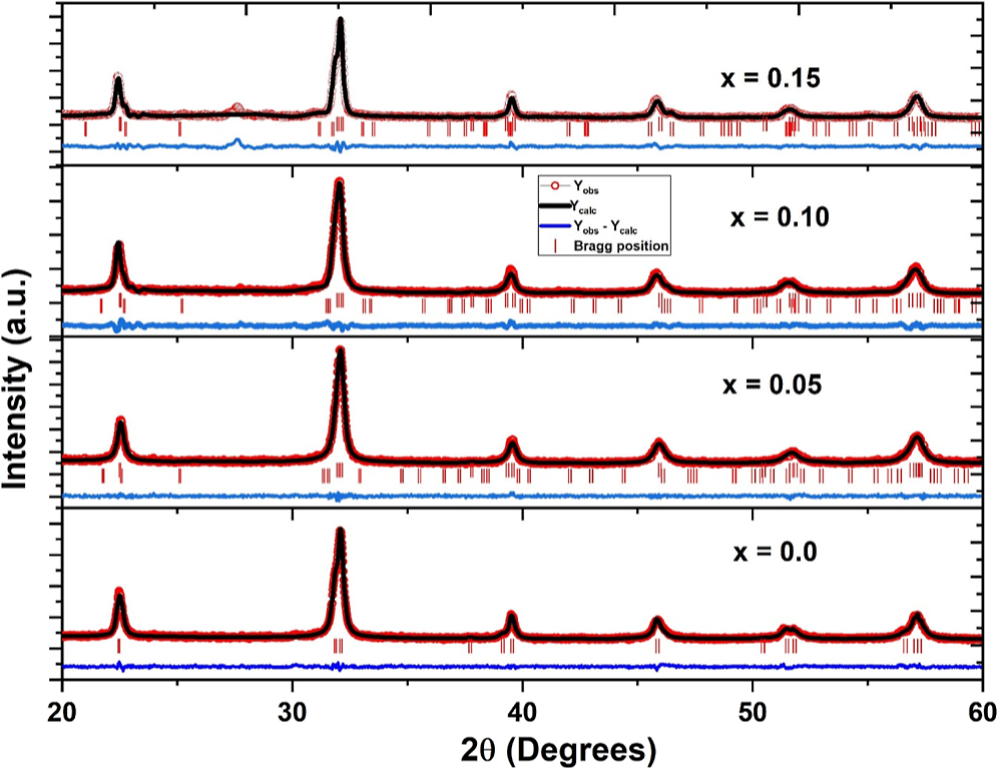
Rietveld refined patterns of Cr^3+^-doped Bi_0.8_Ba_0.1_Pr_0.1_FeO_3_ samples in the composition range 0 ≤ *x* ≤ 0.15.

Figure 1c shows a close look of the 37–60° 2θ range. It is observed that the (024) plane has a near-singlet nature as reported for the *R*3*c* crystal structure, but it changes for higher Cr atom %, and new planes emerge for *x* = 0.10 and 0.15, indicating the coexistence of two crystalline phases. The planes (116) and (112), around 51° in 2θ, also merged and broadened for the higher Cr at. %. Such features in the XRD studies confirm the suggestion of phase coexistence with Cr doping.^27,28^ We employed Rietveld analysis for the XRD patterns of the studied samples to confirm coexistence using the FullProf software with starting fractional coordinates for structural models as enlisted in Table 1.^29^ The starting Rietveld refinement uses a zero-point shift, the unit cell, and background parameters. Figure 2 displays the Rietveld refined patterns for all samples. We test many crystal models to fit the experimental data, but based on the best fit between experimental and calculated data and fitting parameters (see Table 2), the final model for *x* = 0.0 is *R*3*c*, whereas a phase coexistence of *R*3*c* and *Pbnm* symmetries for *x* = 0.05, 0.10, and 0.15 was adopted.

**Table 1:** Atomic Fractional Coordinate for Cr^3+^-Doped Bi_0.8_Ba_0.1_Pr_0.1_FeO_3_ Samples in the Composition Range 0 ≤ *x* ≤ 0.15 in *R*3*c* and *Pbnm* Structural Models

	*R*3*c*				*Pbnm*			
element	site	X	Y	Z	site	X	Y	Z
Bi/Ba/Na	6a	0	0	0.24683(0.25 + s)	4c	0.0003	0.5193	0.25
Fe/Ti	6a	0	0	0.0165 (t)	4a	0	0	0
O1	18b	0.2565	0.3743	0.0910	4c	–0.056	–0.007	0.25
O2					8d	0.2177	0.2802	0.0286

**Table 2:** Extracted Structural Parameters (Phase %, Lattice Parameters, and Fitting Parameters) through Rietveld Analysis, Average Crystallite Size, and Strain for Cr^3+^-Doped Bi_0.8_Ba_0.1_Pr_0.1_FeO_3_ Samples in the Composition Range 0 ≤ *x* ≤ 0.15 in *R*3*c* and *Pbnm* Structural Models

structural model →	*R*3*c* (hexagonal axis) and *P*4*mm*						
parameters	*x* = 0.0 (*R*3*c*)	*x* = 0.05 (*R*3*c*)	*x* = 0.05 (*Pbnm*)	*x* = 0.10 (*R*3*c*)	*x* = 0.10 (*Pbnm*)	*x* = 0.15 (*R*3*c*)	*x* = 0.15 (Pbnm)
phase percentage (%)	100	79.00	21.00	24.32	75.68	25.97	74.03
a (Å)	5.5775	5.5823	*a* = 5.5438	5.5683	*a* = 5.6813	5.5679	*a* = 5.6367
			*b* = 8.1660		*b* = 8.1946		*b* = 8.4553
c (Å)	13.8141	13.7011	5.7084	13.7570	5.4117	13.7590	5.4224
volume (Å^3^)	372.1565	369.7492	258.4231	369.4055	251.9471	369.4015	258.4295
Rp	14.1	13.8	13.8	16.2	16.2	18.3	18.3
Rwp	12.1	12.4	12.4	14.3	14.3	17.5	17.5
R factors	4.67	5.31	5.31	4.87	4.87	4.99	4.99
size (nm) Scherrer’s	28	26	26	27	27	35	35
strain	0.005	0.0120		0.0104		0.0102	

The lattice parameters and fitting parameters are listed in Table 2. It is observed that the unit cell volume decreases with the amount of Cr ions due to the small size of Cr^3+^ (0.615 Å) as compared to that of the Fe^3+^ (0.645 Å) ion, shrinking the unit cell volume. The average crystal size was calculated using the standard Scherrer’s formula
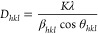
1where *K* represents the shape factor (∼0.9), λ is the used wavelength (1.5405 Å), and β_*hkl*_ is the full width at half-maximum. The calculated average crystallite size is shown in Table 2. It is less than 62 nm, a feature that can be interesting in modifying magnetic properties with Cr doping. On the other hand, the Williamson–Hall (W–H) approach was employed to determine the microstrain (ε) and average crystallite size (*D*) for all studied samples considering only the *R*3*c* structure as per the following formula^30^
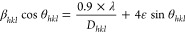
2

**Figure 3:**
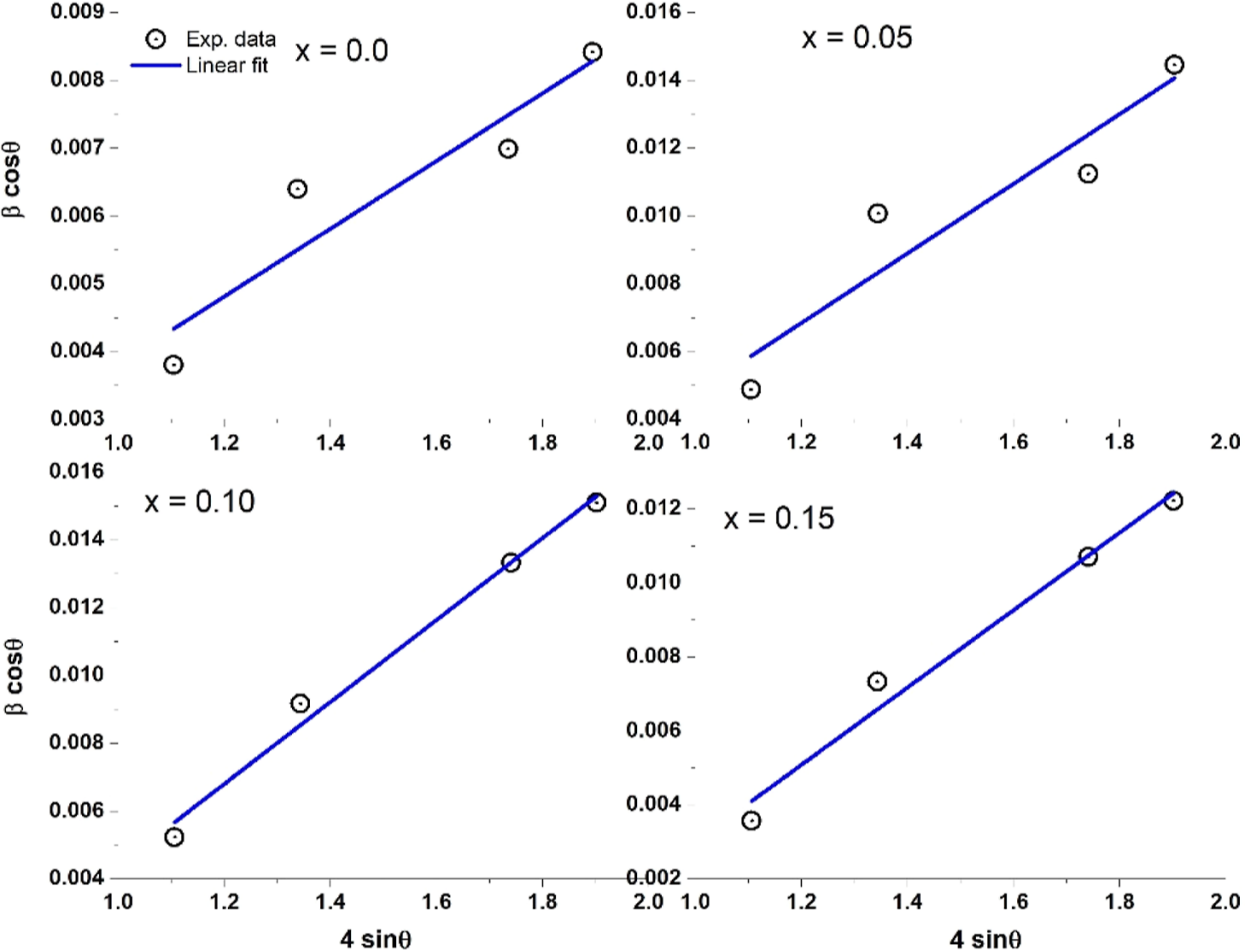
Williamson–Hall (W–H) plots of Cr^3+^-doped Bi_0.8_Ba_0.1_Pr_0.1_FeO_3_ samples in the composition range 0 ≤ *x* ≤ 0.15.

Thus, the β_*hkl*_ cos θ_*hkl*_ vs 4 sin θ_*hkl*_ plots were fitted using a linear fit and are shown in Figure 3. With this, the microstrain and average crystallite size values were calculated and are reported in Table 2. The difference between the average crystallite size obtained by Scherrer’s equation and the W–H approach could be due to the strain and the coexistence of two crystalline phases for Cr-doped samples.

### Optical Properties (UV–Visible)

**Figure 4:**
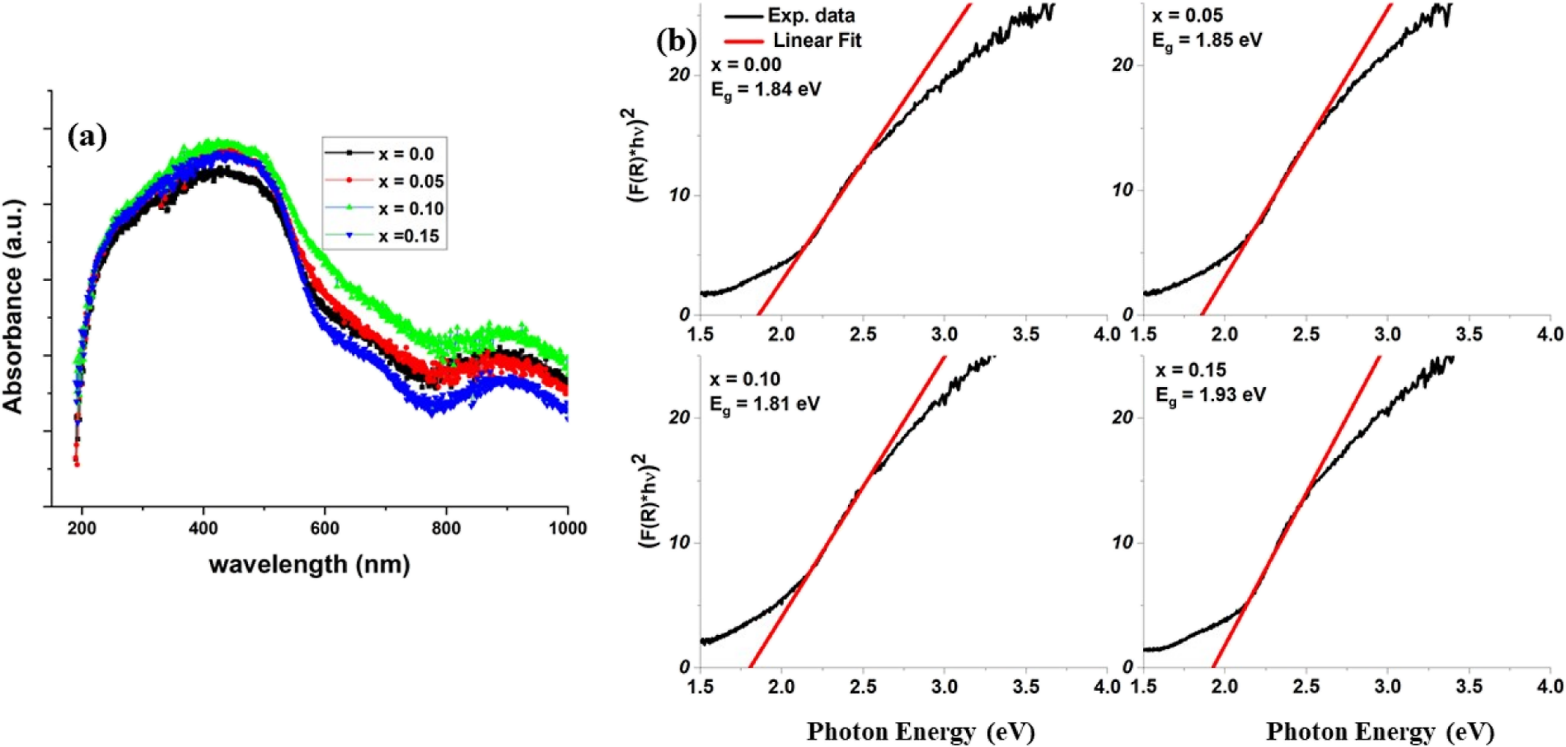
(a) Absorption spectra and (b) [*F*(*R*) × *h*ν]^2^ vs *h*ν plots of Cr^3+^-doped Bi_0.8_Ba_0.1_Pr_0.1_FeO_3_ samples in the composition range 0 ≤ *x* ≤ 0.15.

To explore the optical properties after Cr^3+^ doping, we obtained the absorption spectra for all samples in the 200–1100 nm wavelength range using a UV–visible spectrophotometer at RT. Figure 4a shows the absorption curves for the studied samples. The optical bandgap energy (*E*_g_) was calculated using the Kubelka–Munk function,^31^ denoted as *F*(*R*), and expressed as *F*(*R*) = (1 – *R*)^2^/2*R* = *K*/*S*, where *R* is the relative reflectance, *K* is the absorption coefficient, and *S* is the scattering coefficient. The Kubelka–Munk function can be expressed as [*F*(*R*) × *h*ν]^2^ = *A*(*h*ν – *E*_g_) for direct allowed transitions.^32^ Thus, to determine the bandgap energy, [*F*(*R*) × *h*ν]^2^ vs *h*ν graphs were plotted, and the linear portion of the curves was extrapolated to the *h*ν axis at *F*(*R*) = 0 as shown in Figure 4b.

The obtained direct *E*_g_ values are 1.84, 1.85, 1.81, and 1.93 eV for *x* = 0.0, 0.05, 0.10, and 0.15, respectively. The optical results demonstrate increased bandgap energy values with Cr doping except for *x* = 0.10, which may be attributed to changes in Fe–O bond length and Fe–O–Fe bond angles. Researchers give different explanations for modifying the bandgap in BFO-based materials.^31^ Still, the most reasonable and directly related to the bandgap energy change is the one elaborated around the bond length and angles due to doping. The increase in bandgap energy also seems to be related to crystallite size, as an increase in size increases the bandgap energy of samples.^33^ The observed bandgap energy values lie in the Visible region, a feature that can find applications in photovoltaics and ultrafast optoelectronics devices.^33^ However, it is important to note that the total absorbance (Figure 4a) increases for *x* = 0.10 compared to other Cr doping levels. This suggests that more photons are absorbed by the material at this specific doping percentage within the visible region. As a well-known principle, wider band gap materials tend to absorb fewer photons than those with smaller band gaps, establishing an inverse relationship between absorbance and band gap. In the present case, for *x* = 0.10, this relationship holds true. The decrease in band gap can also be attributed to Cr ions creating allowed states near the valence or conduction bands, effectively reducing the material’s band gap.^34,35^

### FTIR Analysis

FTIR spectroscopy was employed to analyze the vibrational dynamics and associated rotational–vibrational bands of the samples, shedding light on the chemical and structural modifications in BiFeO_3_ induced by doping.

**Figure 5:**
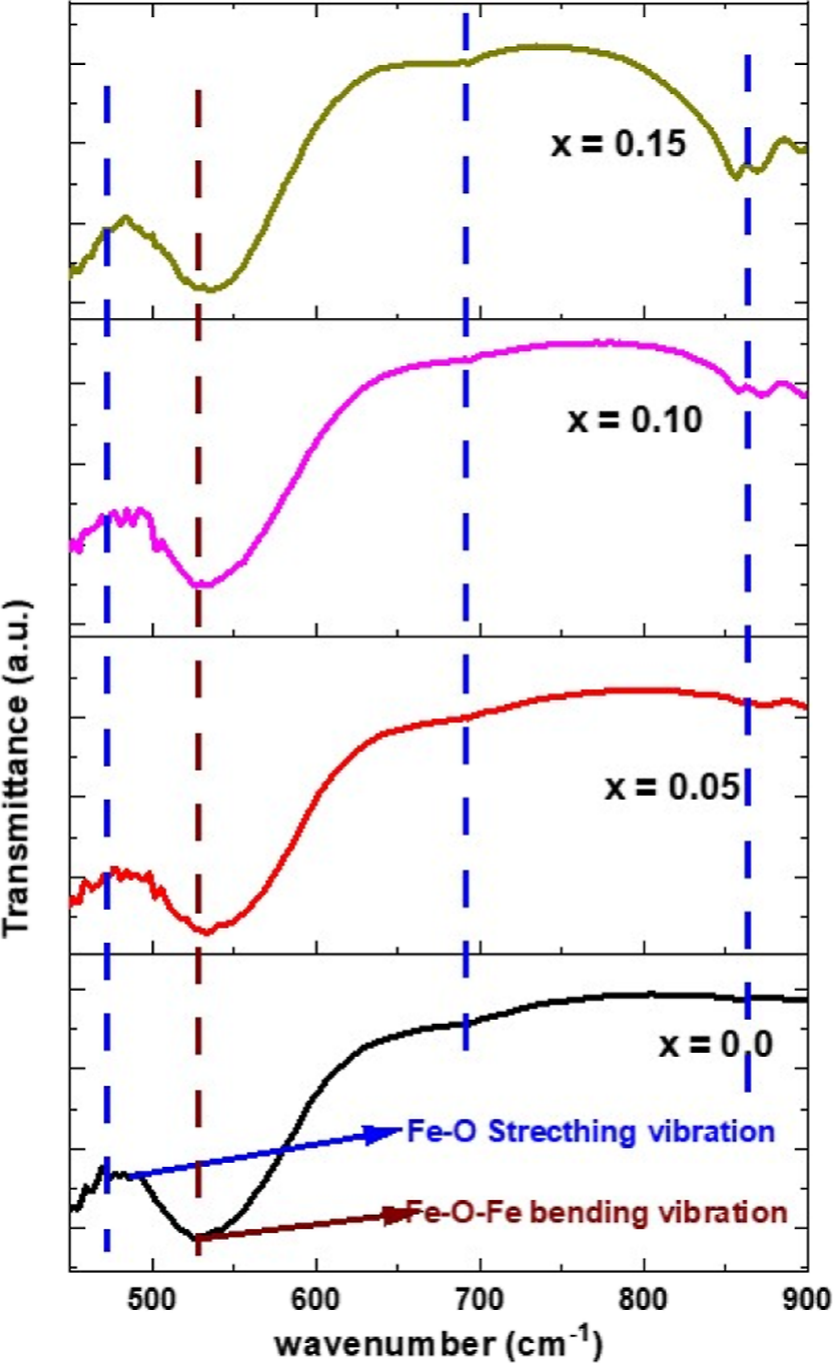
FTIR spectra of Cr^3+^-doped Bi_0.8_Ba_0.1_Pr_0.1_FeO_3_ samples in the composition range 0 ≤ *x* ≤ 0.15.

As depicted in Figure 5, the FTIR spectra of all samples exhibit IR-active optical phonon modes, characterized by a band within the 400–800 cm^–1^ range. Notably, minor absorption bands near 443 cm^–1^ are discernible for all samples, ascribed to the Fe–O bending vibrations.^36,37^ A pronounced broad hump around 525 cm^–1^, slightly shifted toward higher wavenumbers due to the disparate ionic sizes of dopants and host, is observed across all samples. This feature is attributed to the confluence of Fe–O and Bi–O stretching and bending vibrations within the BO_6_ octahedra of the perovskite structure.^37^ Additionally, a small hump near 700 cm^–1^ is linked to the Bi–O stretching and bending vibration.^37−41^ The hump at approximately 865 cm^–1^ is indicative of the entrapped NO_3_ ions. The presence of a metal-oxide band within the 400–700 cm^–1^ interval corroborates the formation of the perovskite structure, a finding supported by other BFO-based material studies.^37−41^ Furthermore, Ke et al. have identified the absorption bands in the 400–600 cm^–1^ spectral region as fundamental peaks for metal oxides, confirming the successful synthesis of BFO perovskite materials.^37−41^

### Dielectric Properties

**Figure 6:**
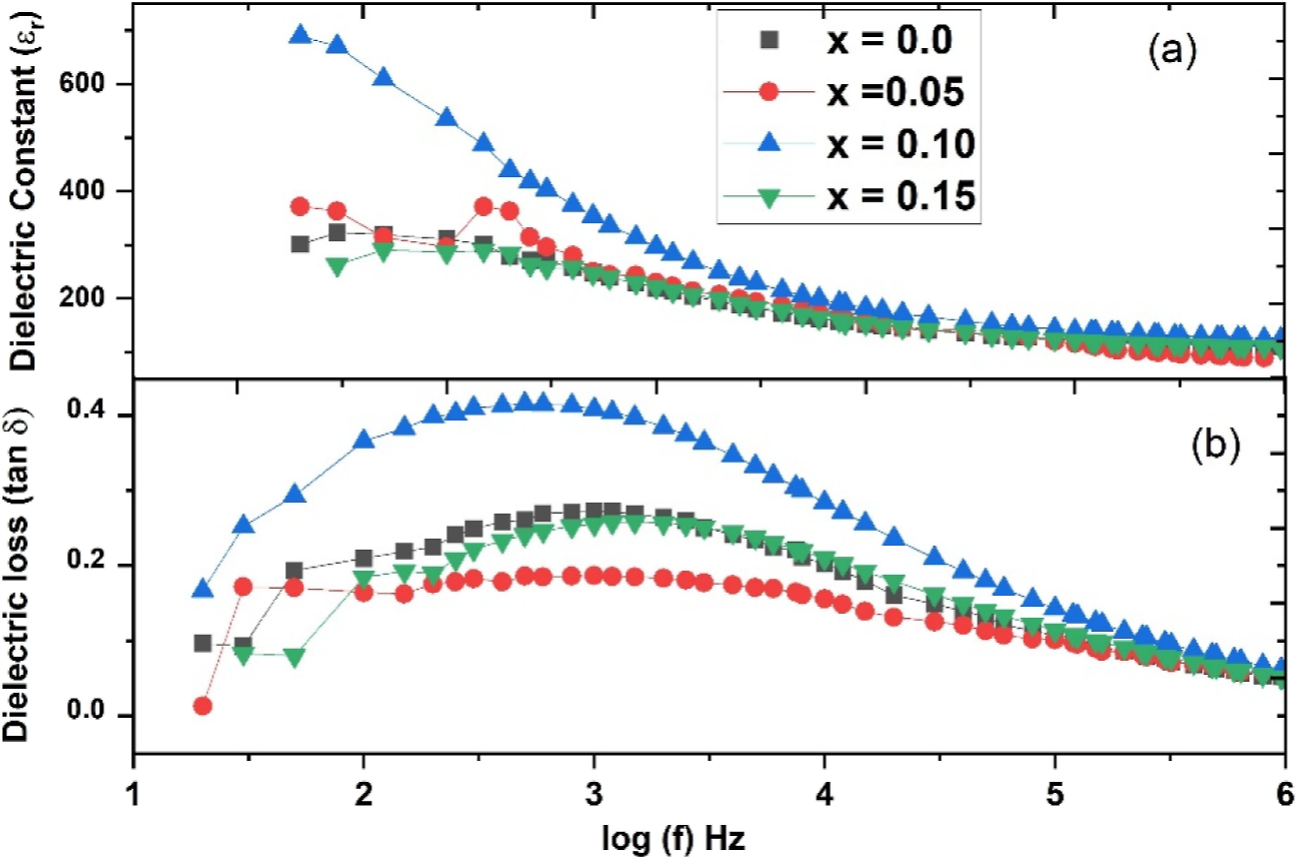
RT dielectric properties: (a) dielectric constant and (b) dielectric loss of Cr^3+^-doped Bi_0.8_Ba_0.1_Pr_0.1_FeO_3_ samples in the composition range 0 ≤ *x* ≤ 0.15.

Dielectric measurements were performed on sintered ceramics to explore the impact of Cr ion doping at RT for all samples. Figure 6 shows the plots of the dielectric constant (ε_r_) and dielectric loss (tan δ) as a function of frequency. The dielectric constant decreases with increasing frequency, indicating a typical dielectric behavior, followed by temperature-independent behavior for higher frequencies.^27^

It is noted that the dielectric constant first increases with Cr ion doping and then decreases again. Interestingly, the dielectric loss at a lower frequency shows a broad hump and then decreases with an increase in frequency, the same as the dielectric constant. These samples show a lower frequency dispersion (i.e., frequency-dependent dielectric constant) in agreement with Koop’s theory, which states that electric dipoles need some time to align with an applied electric field.^21,27,28^ Moreover, at a frequency of 1 kHz, we observed that the dielectric constant escalates for Cr concentrations of (*x* = 0.05) and (*x* = 0.10), while it diminishes for (*x* = 0.15). The dielectric loss exhibits a similar pattern and remains commendably below 0.4 for all samples, which bodes well for the performance of BFO-based electronic devices. The enhancements in dielectric constant and loss at 1 kHz, as compared to non-Cr-doped specimens, are attributed to the reduction of oxygen vacancies that arise from charge compensation when Cr ions are substituted for Fe in the BO_6_ octahedra.^15,27,28^ Additionally, we note that the dielectric constant initially increases with Cr doping in the low-frequency domain but then decreases; conversely, it remains relatively unchanged in the high-frequency domain. Introducing Cr into the BFO lattice is expected to generate a modest number of defects, thereby increasing the dielectric polarization and the dielectric constant. However, as the Cr concentration continues to rise, the proliferation of crystal defects leads to a reduction in dielectric polarization and dielectric constant. Moreover, structural phase transitions also significantly influence the material’s polarization. In this study, the orthorhombic phases exhibit an increase up to a Cr concentration of (*x* = 0.10) and then a decrease at (*x* = 0.15).

### Magnetic Analysis

**Figure 7:**
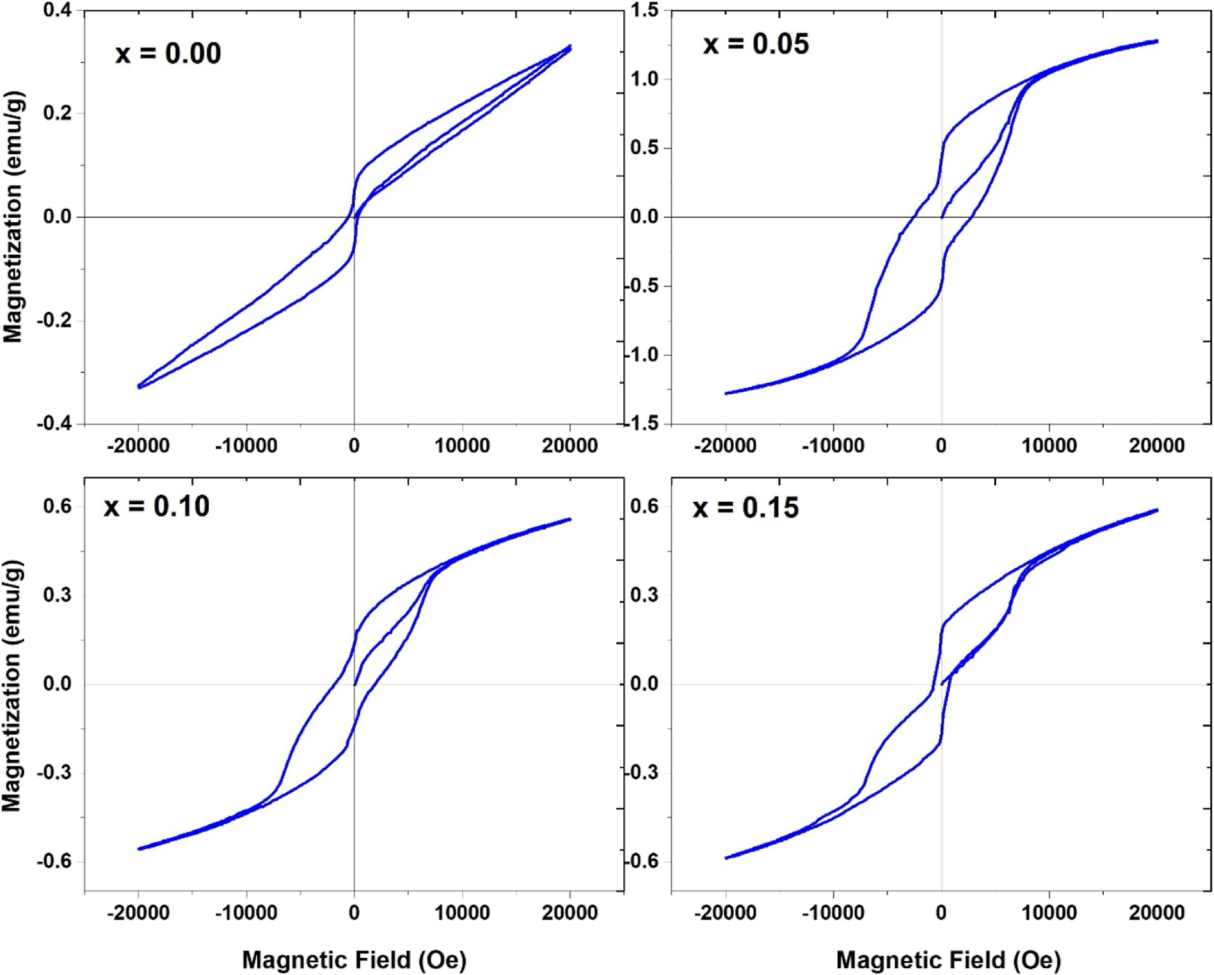
Magnetic properties (M–H loops) at RT of Cr^3+^-doped Bi_0.8_Ba_0.1_Pr_0.1_FeO_3_ samples in the composition range 0 ≤ *x* ≤ 0.15.

Ion substitution is an important technique to modify physical properties, particularly magnetic properties, when doping with transition metals. We measured magnetization as a function of the magnetic field (M–H curves) to investigate the impact of Cr^3+^ ions on the magnetic properties of BBPFO ceramics, as shown in Figure 7. Interestingly, Pr^2+^ and Ba^2+^ doping promotes weaker ferromagnetic ordering in the BFO lattice than undoped BFO materials due to the structural distortions caused by Pr and Ba. Pure BFO presents a G-type antiferromagnetic ordering. Furthermore, the magnetic moment of Fe^3+^ ions can align in ferromagnetic ordering with that of Pr^3+^. The average crystallite size (26–35 nm in the present case) also improves the overall magnetization by breaking the 62 nm spin spiral structure of BFO.^27^ As Cr^3+^ ions substitute for Fe, the magnetization increases, approaching saturation; however, saturation is not achieved completely due to the AFM nature of the BFO lattice.

Such improvement in magnetic properties with adding Cr^3+^ ions substituting for Fe^3+^ ions is due mainly to two factors. The first one is the difference in the magnitude of the magnetic moments of Cr^3+^ (3.87 μ_B_) and Fe^3+^ (5.92 μ_B_) in the octahedral sites, which leads to uncompensated spins in the BFO lattice and results in an increase in the overall magnetization.^42^ The other possible reason for the increased magnetization with Cr ion doping is the strong ferromagnetic coupling through superexchange interaction in the form of local ferromagnetic supercells because of the different ionic sizes of Fe^3+^ and Cr^3+^.^43,44^ Notably, the remnant magnetization decreases for *x* = 0.10 and 0.15 compared to *x* = 0.05, possibly due to the increased fraction of the orthorhombic phase in these samples, as reported by Kumar et al.^44^ It can be seen that the coercivity is increasing with 5 at. % doping and then decreasing up to 15 at. %. It is important to note that we have achieved a low value of coercivity and a high value of remnant magnetization compared to the available reported values in the literature.^45,46^ This result is very important in memory applications and is shown in Figure 7 and Table 3. The maximum magnetization is achieved for the 5 at. % Cr doping, as shown in Figure 7.

**Table 3:** Experimental Magnetic Parameters (*M*_r_, *M*_s_, and *H*_c_), Squareness Ratio (*S*), and AFM Contribution **(**χ_M_) for Cr^3+^-Doped Bi_0.8_Ba_0.1_Pr_0.1_FeO_3_ Samples in the Composition Range 0 ≤ *x* ≤ 0.15 in *R*3*c* and *Pbnm* Structural Models

Cr-doped BBPFO	magnetic and ferroelectric experimental parameters				
	*M*_r_ (10^–3^) (emu/g)	*M*_S_ (10^–3^) (emu/g)	*H*_C_ (Oe)	χ_M_	*S* = *M*_r_/*M*_s_
*x* = 0.0	0.053	0.32	353	1.25 × 10^–5^	0.16
*x* = 0.05	0.454	1.27	2835	1.63 × 10^–5^	0.35
*x* = 0.10	0.14	0.55	1866	2.13 × 10^–7^	0.25
*x* = 0.15	0.18	0.58	800	5.16 × 10^–6^	0.31

**Figure 8:**
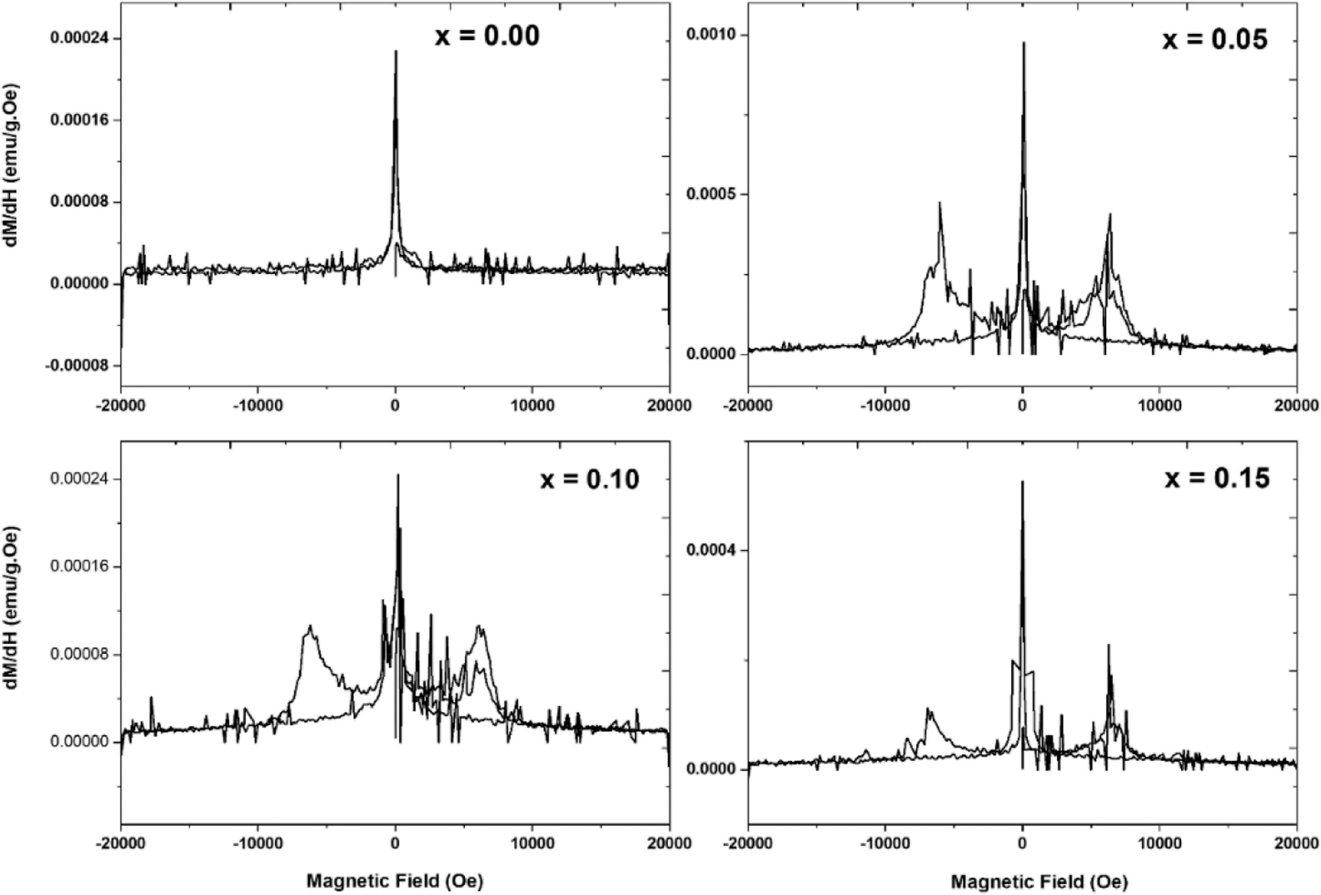
dM/dH plots of Cr^3+^-doped Bi_0.8_Ba_0.1_Pr_0.1_FeO_3_ samples in the composition range 0 ≤ *x* ≤ 0.15.

Figure 8 shows the dM/dH vs H plots for all of the compositions. It displays the exact coercivity values in Table 3. The critical magnetic field is determined from the maxima of dM/dH vs H plots, which symbolizes dynamical magnetic alterations surrounding the present systems. The exact value of the magnetic field (i.e., coercivity) conforms to the maxima of dM/dH for both the positive and negative cycles, i.e., slight half-width. It fluctuates when the Cr percentage in the host system changes and moves toward a higher exact magnetic field with respect to the improved Cr content (shown in Figure 7).^47^

The squareness (*S*) is calculated by the ratio of the remnant magnetization to the saturation magnetization.^46^ The maximum value of *S* is 1, which suggests using material in-memory applications. In the present case, we have calculated the *S* values for all the compositions, tabulated in Table 3. As far as BiFeO_3_ materials are concerned, we have achieved good *S* values in the range of 0.16–0.35.^46^

One important result of the investigation can be seen in Figure 7 for 15 at. % of Cr doping, showing low coercivity and high maximum magnetization. Such excellent characteristics of the hysteresis loop may be useful for memory applications. In addition to the low coercivity and high magnetization, this composition has an excellent squareness ratio of ∼0.31, proving its usefulness in-memory applications. A detailed study of this composition is being processed.

**Figure 9:**
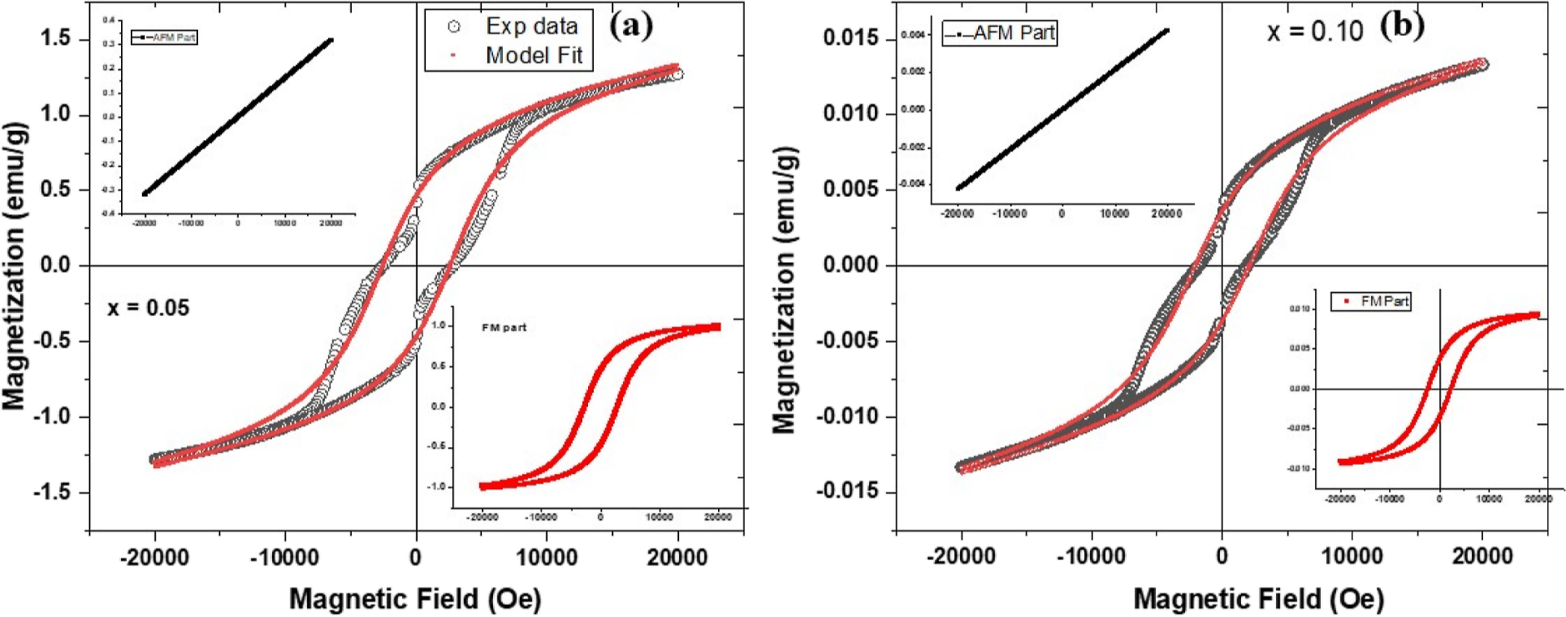
Deconvoluted M–H curves of the ferromagnetic contribution of the typical samples (a) *x* = 0.05 and (b) *x* = 0.10. In both figures, the upper left inset shows the AFM contribution and the lower right inset shows the ferromagnetic contribution.

To explore the magnetic behavior and the contribution of ferromagnetic and antiferromagnetic/paramagnetic contribution in the M–H plots of these samples, we have carried out theoretical fitting to the experimental data as per the following equation^47^
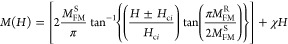
3Here, χ is the magnetic susceptibility of the antiferromagnetic part. In the first term, *M*_FM_^S^ is the ferromagnetic (FM) saturation magnetization, *M*_FM_^R^ is the remnant magnetization, and *H*_ci_ is the intrinsic coercivity. Figure 9 shows the fitted M-H loops with experimental data with the FM and AFM contributions for the *x* = 0.05 and 0.10 samples. A similar approach was adopted to analyze the other compositions, and all parameters are presented in Table 3. It is observed that from Figure 9, there is a best fit between the model and the experimental data for *x* = 0.05 and 0.10. The *M*_r_, *M*_s_, and *H*_c_ were found to follow the same trend as in the experimental values for these samples.

## Conclusions

In essence, we have successfully synthesized polycrystalline Cr ion-doped Bi_0_._8_Ba_0_._10_Pr_0_._10_FeO_3_ materials via the sol–gel method. We thoroughly examined and discussed their structural, optical, and multiferroic properties. Through Rietveld structural analysis, the rhombohedral (*R*3*c*) phase at (*x* = 0.0) was validated while doping with Cr^3+^ ions induced a concurrent orthorhombic phase alongside the rhombohedral phase across all doped specimens. Optical studies have revealed a Cr concentration-dependent shift in bandgap energy from 1.84 eV (*x* = 0.0) to 1.93 eV (*x* = 0.15) in the bandgap energy values situated in the Visible spectrum, hinting at potential photovoltaic device applications. Dielectric response enhancements were noted upon Cr^3+^ ion substitution. Moreover, magnetic property improvements were observed with Cr ion doping in comparison to undoped Bi_0_._8_Ba_0_._10_Pr_0_._10_FeO_3_ samples, with remnant magnetization (*M*_r_) increasing from (0.053 × 10^–3^) emu/g for *x* = 0.0 to *M*_r_ = 0.18 × 10^–3^ emu/g for *x* = 0.15. The advancements in multiferroic and optical properties observed in this study are promising and may pave the way for novel applications in photovoltaic and memory storage technologies.
